# Impact of early fructose intake on metabolic profile and aerobic capacity of rats

**DOI:** 10.1186/1476-511X-10-3

**Published:** 2011-01-11

**Authors:** Ana C Ghezzi, Lucieli T Cambri, Carla Ribeiro, José D Botezelli, Maria AR Mello

**Affiliations:** 1 São Paulo State University - UNESP Department of Physical Education Av: 24-A, 1515 Bela Vista Zip code: 13506-900 Rio Claro - São Paulo- Brazil

## Abstract

**Background:**

Metabolic syndrome is a disease that today affects millions of people around the world. Therefore, it is of great interest to implement more effective procedures for preventing and treating this disease. In search of a suitable experimental model to study the role of exercise in prevention and treatment of metabolic syndrome, this study examined the metabolic profile and the aerobic capacity of rats kept early in life on a fructose-rich diet, a substrate that has been associated with metabolic syndrome.

**Methods:**

We used adult female Wistar rats fed during pregnancy and lactation with two diets: balanced or fructose-rich 60%. During breastfeeding, the pups were distributed in small (4/mother) or adequate (8/mother) litters. At 90 days of age, they were analyzed with respect to: glucose tolerance, peripheral insulin sensitivity, aerobic capacity and serum glucose, insulin, triglycerides, total cholesterol, LDL cholesterol and HDL cholesterol concentrations as well as measures of glycogen synthesis and glucose oxidation by the soleus muscle.

**Results:**

It was found that the fructose rich diet led the animals to insulin resistance. The fructose fed rats kept in small litters also showed dyslipidemia, with increased serum concentrations of total cholesterol and triglycerides.

**Conclusion:**

Neither the aerobic capacity nor the glucose oxidation rates by the skeletal muscle were altered by fructose-rich diet, indicating that the animal model evaluated is potentially interesting for the study of the role of exercise in metabolic syndrome.

## Background

Clinically, the metabolic syndrome, also known as X syndrome or insulin resistance syndrome comprises a spectrum of disorders in which impaired glucose tolerance represents one of the most important. These changes include insulin resistance, with or without type 2 diabetes mellitus, hypertension, obesity, dyslipidemia, endothelial dysfunction, among others [1].

It is estimated that the prevalence of metabolic syndrome is 24% of the adults and between 50-60% of the population over 60 years in the United States. In 2002, only in this country, according to estimations, there were more than 47 million people with manifestations of this syndrome [2].

The identification of the metabolic syndrome is controversial because there is no single international criterion with the definitive description. Reaven [3] suggested a strong association between individuals with the same cardiovascular risk factors and designated "X syndrome". Their common denominator was represented by insulin resistance. At the time he, proposed five consequences, all at high risk for cardiovascular disease: glucose intolerance, hyperinsulinemia, increased serum triglycerides, decreased serum HDL cholesterol and hypertension. Obesity and physical inactivity increase insulin resistance and thus aggravate the syndrome. However, this syndrome can be found in healthy individuals, with body weight and normal glucose tolerance [4].

According to Duncan & Schmidt [5], metabolic syndrome is characterized by a cluster of risk factors for cardiovascular disease and diabetes, usually linked to insulin resistance and central obesity. Recently, the European Association for the Study of Diabetes (EASD), in conjunction with the American Diabetes Association published positions about the metabolic syndrome. According to these associations, the metabolic syndrome, which is regarded as a predictor of cardiovascular disease, is poorly defined, requiring more research to help understanding more adequate procedures for its treatment [6,7]. Also according to these associations, the metabolic syndrome is generally defined by the presence of three or more of the following characteristics: high waist circumference, increased blood concentrations of triglycerides, high blood pressure, low circulating HDL cholesterol and high blood glucose concentrations [6,7].

The World Health Organization (WHO) offers a slightly different definition, including anyone who has diabetes or insulin resistance and two of the following characteristics: high waist-hip ratio, high serum concentrations of triglycerides or low serum HDL cholesterol concentration, high blood pressure and high urinary albumin excretion [8]. According to Gale [6] and Kahn [7], taken individually, each of the aforementioned conditions is considered as a risk factor for cardiovascular disease and should be treated as such. All together, these observations show the urgency to expand the studies on the metabolic syndrome.

There are clinical and epidemiological evidence suggesting an association between the progressive development of metabolic syndrome and high consumption of fructose [9]. Indeed, a significant increase in the prevalence of obesity and metabolic syndrome has been linked to a 30% increase in total intake of fructose in the last 20 years in the United States. This has been coupled with the introduction of corn syrups and sweeteners in soft drinks and other foods with high amounts of fructose [10].

Moderate hypertension, glucose intolerance, insulin resistance, hyperinsulinemia and hypertriglyceridemia, which are signs of metabolic syndrome, were induced in rats by prolonged feeding with high-fructose diets [11,12]. Therefore, rats fed with this type of diet have been widely used as an experimental model of human metabolic syndrome [13-15]. However, there are studies showing that in the absence of other dietary manipulations, fructose-fed rats do not develop the complete picture of the disease [12]. Also, there are still discrepancies in the literature regarding the changes in glucose homeostasis by administration of fructose-rich diets. Some studies state that these changes occur [16-19], but others do not confirm [20-22]. Therefore the study of the factors that characterize fructose-induced metabolic syndrome in rodents deserve more attention.

It is also known that poor nutrition *in uterus *can "program" the fetal tissues in order to make them more vulnerable to food-related disorders such as type 2 diabetes, metabolic syndrome and chronic degenerative diseases in adulthood [23]. Considering that studies using animals maintained on fructose-rich diets as an experimental model of metabolic syndrome, administer the diet after birth, at different periods of life, is of interest to determine the metabolic effects of high fructose doses consumed *in uterus*.

Moreover, it was shown that the amount of food consumed during breast feeding has an important role in determining the feeding behavior in adulthood. In a study with rats kept during lactation in litters of 4, 13, 17 or 22 pups per mother, the litter size was inversely associated with dietary intake in adulthood [24]. According to the authors, this shows that the control of food intake of newborn rats permits "programming" of food intake later in life. Therefore, it is also of interest to manipulate the amount of food offered to the neonate animal undergoing treatment with fructose.

In the treatment of metabolic syndrome, exercise has been considered of great importance [25]. Such an intervention demonstrated to improve glucose tolerance and to attenuate insulin resistance. Since there are limitations in human research, animal models offer more suitable conditions to study the issue. Therefore, it seems of importance to verify if the feeding protocols imposed to rats early in life interfere with the ability to perform physical exercise in adulthood.

In search of a suitable experimental model to study the role of exercise in the treatment of metabolic syndrome, this study examined the metabolic profile as well as glucose uptake by skeletal muscle and aerobic capacity of rats maintained on a fructose-rich diet during intrauterine and postnatal life.

## Materials and methods

### Animals and treatment

Adult pregnant Wistar (90 days) rats, fed during pregnancy and lactation with two diets: balanced (AIN-93G) or fructose-rich (60% fructose) were used. During breastfeeding, the pups were distributed in small (4/mother) or adequate (8/mother) litters. After weaning, they were kept in the same diets until 90 days. All animals had body weight and length (nose to anus) and food intake recorded once a week from weaning (21 days) on. All procedures used with animals were approved by the Ethics Committee in Animal Experimentation/CEEA, Campinas State University (UNICAMP), under the Protocol 1487-1.

### Diet treatment

During the experiment, food consisted of balanced or fructose-rich semi-purified diets, as described in Table 1.

**Table 1:** Diet treatment

Components (g/kg)	**Balanced**^**1**^	Fructose-rich (60%)
**Casein**	202	202
**Com Starch**	397	-
**Dextrin**	130.5	-
**Sucrose**	100	27.6
**Fructose**	-	600
**L-cystine**	3	3
**Soybean oil**	70	70
**Mineral mix (AIN-93GMX)**^**1**^	35	35
**Vitamin mix (AIN-93GVX)**^**1**^	10	10
**Fiber**	50	50
**Choline chloridrate**	2.5	2.5

1. According to the Reeves [26].

### Experimental Groups

1. Control (C): rats maintained on the balanced diet in intrauterine and postnatal life, nursed in appropriate litters (n = 7);

2. Control/Small litter (CS) rats maintained on the balanced diet in intrauterine and postnatal, nursed in small litters (n = 7);

3. Fructose (F) rats maintained on the high-fructose diet in intrauterine and postnatal life, nursed in appropriate litters (n = 7);

4. Fructose/Small litter (FS) rats maintained on the high-fructose diet in intrauterine and postnatal life, nursed in small litters (n = 7);

### Glucose tolerance test - GTT

This test was performed with the animals at the end of the experiment, after 12 hours of fasting. A first blood sampling was performed through a small cut on the tip of the tail (time 0). Afterwards, an 80% glucose solution (2 g/kg of weight) was administered to the rats through a polyethylene gastric probe. Blood samples were collected after 30, 60 and 120 minutes with heparinized capillaries and calibrated to 25 μL, for of glucose and insulin determinations. The blood glucose concentrations were determined by the glucose oxidase-peroxidase enzymatic colorimetric method using commercial kits (LABORLAB^®^) and the blood insulin concentrations by ELISA, also employing commercial kits (SIGMA^®^). The area under the serum glucose curve during the test were evaluated using the trapezoidal method (AG = mg*120 min) with ORIGIN PRO 8 software.

### Insulin tolerance test - ITT

Insulin sensitivity was evaluated by the insulin tolerance test. The test consisted of a bolus injection of insulin (300 mU/kg body weight) followed by blood sample collections, for the measurement of glucose concentrations, from a cut at the tip of the tail before and 30, 60 and 120 minutes after the insulin injection. The serum glucose disappearance rate (Kitt) was calculated using the formula 0.693/*t*_1/2 _where *t*_1/2 _is the half-life of the process. The serum glucose half-life was calculated from the slope of a least-square analysis of serum glucose concentrations from 0 to 60 minutes after the subcutaneous injection of insulin, during this time, the glucose reduces linearly [27].

### Maximal Lactate Steady State - MLSS

The identification of the aerobic/anaerobic metabolic transition during swimming was performed using the MLSS protocol. The first swimming test was started 48 hours after the completion of the ITT. In short, the animals were submitted to four swimming tests of increasingly intensities in which they supported constant workloads relative to body weight in each test. These tests were given at intervals of forty eight hours until the stabilization of blood lactate concentrations during exercise was no longer possible. Each test consisted of thirty minutes of continuous swimming supporting the chosen workload, with blood collection by a small cut at the tip of the tail every five minutes to determine the concentrations of lactate. The blood lactate concentrations were then determined by a spectrophotometer [28]. The criterion used for stabilization was a difference less than or equal to 1.0 mM of blood lactate between the 10^th ^and 25^th ^minutes of exercise [29].

### Blood and Tissue sample collection

At the end of the experiment, the animals were killed by decapitation, 48 hours after the last *in vivo *evaluation, at rest, the blood being collected for the determination of serum concentrations of glucose, triglycerides, total cholesterol, LDL cholesterol, HDL cholesterol by colorimetric methods, using commercial kits (LABORLAB^®^) and of insulin by ELISA, also using commercial kits (SIGMA^®^).

The adipose tissue of the posterior subcutaneous, mesenteric and retroperitoneal regions were removed and weighed for determination of total lipids content. Excision of the different fat deposits was carried out according to the description of Cinti [30] The total lipid concentrations were determined by the procedure described by Nogueira [31].

For the assessment of glucose metabolism, soleus muscle longitudinal strips weighing 25 - 35 mg (wet weight) were first incubated for 30 min at 37°C in a Dubinoff water bath, inside glass scintillation vials containing 1.5 mL of a Krebs bicarbonate buffer (NaCl 0.6%; HEPES 6.64 mM; KCl 0.032%; CaCl_2 _1.14 mM; KH_2_PO_4_0.015%; NaHCO_3 _0.19%; MgSO_4 _0.03%) equilibrated with a mixture of 95%O_2_-5%CO_2_, pH 7.4. After this, the muscle strips were transferred to new glass scintillation vials (outer vials) containing 1.5 mL of Krebs-bicarbonate buffer supplemented with glucose 5.5 mM, containing [U^14^C]glucose (0.25 mCi/mL) and [3H+] 2-deoxiglucose (2DG, 0.5 μCi/mL) and insulin (100 μU/mL). Inside these scintillation vials, other glass vials (inner vials), which were formed like a scoop with an upwards-directed straight shaft, containing 700 mL of hyamine 10-x were installed. The shafts of the inner vials were squeezed about 1 cm through a small hole in a round rubber membrane. The outer vials were sealed with the rubber membrane and lacked with plastic rings. This system, containing the muscle strips, was incubated in the Dubinoff water bath for 60 min. The release of CO_2 _was stimulated by the injection of 200 μL of trichloroacetic acid 25% into the outer vials and the CO_2 _was trapped in hyamine 10-x during a further 3 h incubation at 37°C. Glucose incorporation to glycogen (glycogen synthesis) was determined by measuring the radioactivity of the ^14^C in the precipitated obtained during muscle glycogen extraction process [32]. Glucose oxidation was estimated by the measurement of the radioactivity of the ^14^C in the inner vial liquid. Glucose (2DG) uptake was evaluated in the alkaline phase obtained during muscle glycogen extraction process, by measuring the radioactivity of the 3H+. All measurements of radioactivity were carried out in a PACKARD Tricarb 2100 scintillation counter, in a TRITON X-100 toluene-based scintillant

### Statistics

The results were analyzed by analysis of variance (ANOVA) and, when necessary, by post hoc Newman-Keuls. In all cases, the level of significance was set at 5%. The software used for the analysis was Statistica 7.0.

## Results

**Figure 1:**
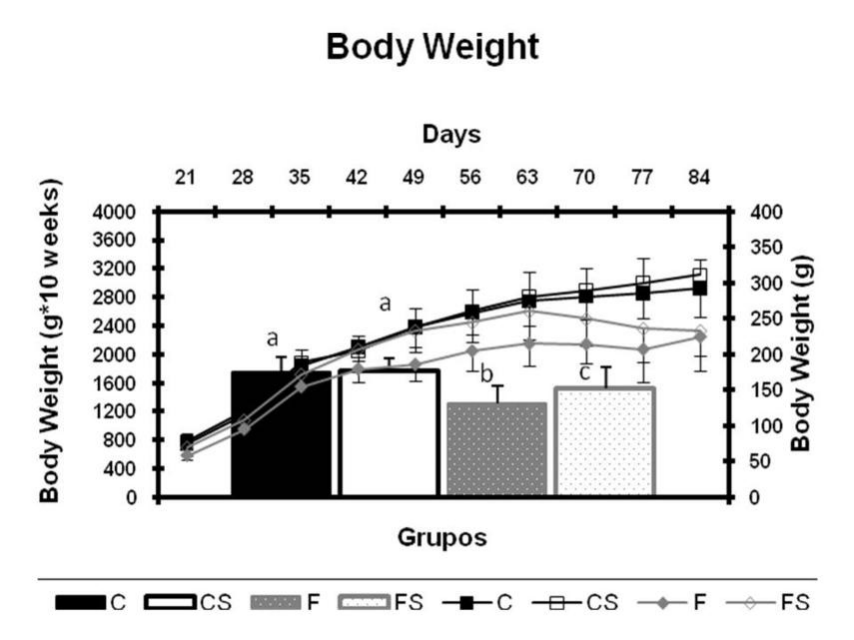
**Body Weight and Area Under the Curve of Body Weight** (A) Body weight (g) and (B) area under the curve of body weight (g.10 weeks) during the experiment. C = balanced diet, adequate litter; CS = balanced diet, small litter, F = fructose-rich diet, adequate litter, FS = high-fructose diet, small litter. Results are mean ± SD of 7 animals per group. Different letters indicate statistically significant differences (Two-way ANOVA and Newman-Keuls post hoc, P < 0.05).

Figure 1 (A) reveal the evolution of animals' body weight throughout the experiment. Data analysis was performed by the area under the curve of body weight (B). The groups fed with the fructose-rich diet (F and FS) had lower body weight than controls, with the lowest values given by the group kept in appropriate litters during lactation (F).

**Figure 2:**
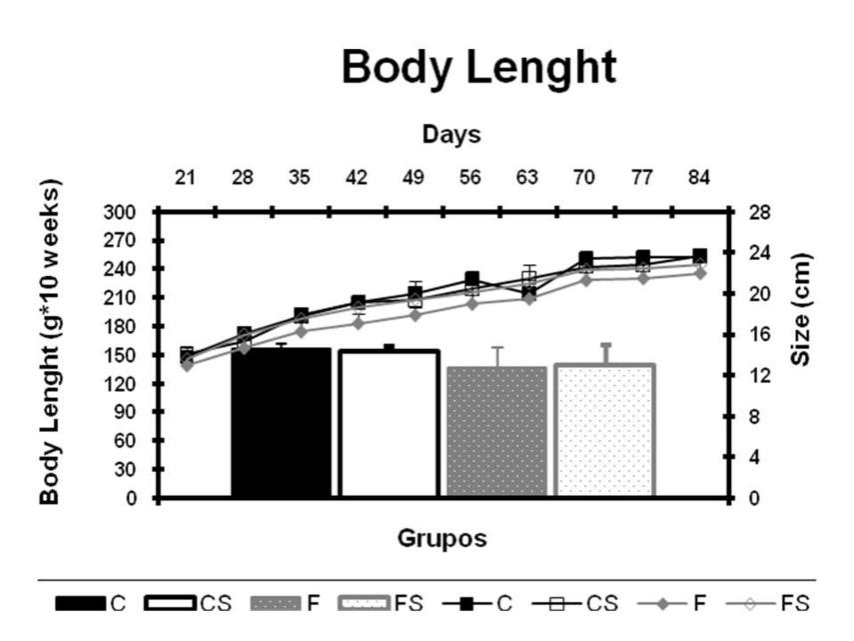
**Nose-Anus Length and Area Under the Curve of Nose-Anus Length** (A) Nose to anus length (cm) and (B) area under the curve of nose-anus length (cmx10 weeks) from weaning (21 days) to the end of the experiment (90 days). C = balanced diet, adequate litter; CS = balanced diet, small litter, F = fructose-rich diet, adequate litter, FS = high-fructose diet, small litter. Results are mean ± SD of 7 animals per group.

Figure 2: Presents the results of the nose-to-anus length of the animals (A). Data analysis was performed by the area under the curve of nose-to-anus length (B). No significant difference was found between the groups.

**Figure 3:**
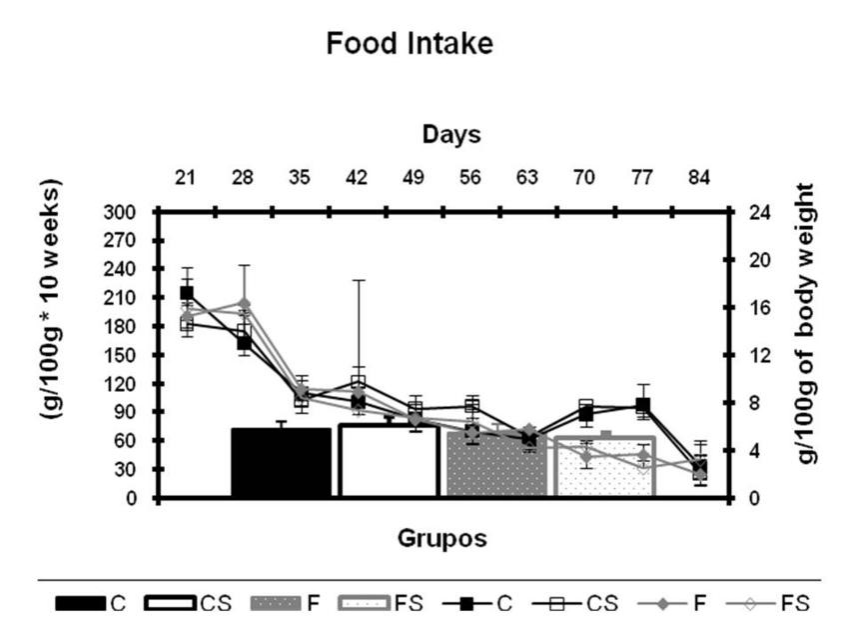
**Food Intake and Area Under the Curve of Food Intake** (A) Food intake per 100 g of body weight (g/100 g) and (B) area under the curve of food intake (g/100 g. 10 weeks) weight body from weaning (21 days) to the end of the experiment (90 days). C = balanced diet, adequate litter; CS = balanced diet, small litter, F = fructose-rich diet, adequate litter, FS = high-fructose diet, small litter. Results are mean ± SD of 7 animals per group.

Figure 3 shows the food intake per 100 g of body weight and per day of the animals during the experiment (A). Data analysis was performed by the area under the curve of food intake (B) of the animals. No significant difference was found between the groups.

**Figure 4:**
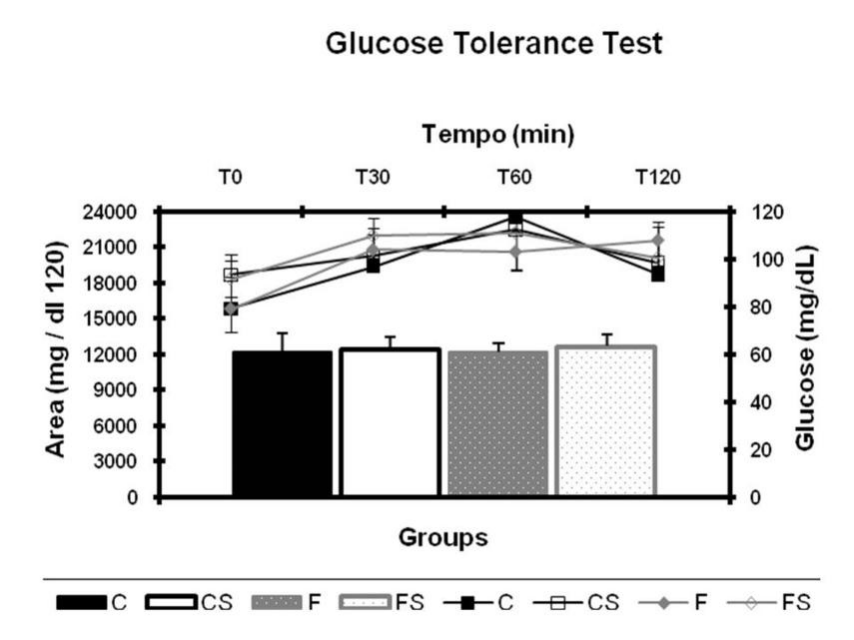
**Glucose Kinetics and Area Under Curve of Glucose During OGTT** (A) Serum glucose (mg/dl) and (B) area under serum glucose curve (mg/dl.120) during the oral Glucose Tolerance Test (GTT). C = balanced diet, adequate litter; CS = balanced diet, small litter, F = fructose-rich diet, adequate litter, FS = high-fructose diet, small litter. Results are mean ± SD of 7 animals per group.

Figure 4 contains the blood glucose concentrations data (A) and area under blood glucose curve (B) during the glucose tolerance test (GTT). No significant difference was found between the groups.

**Figure 5:**
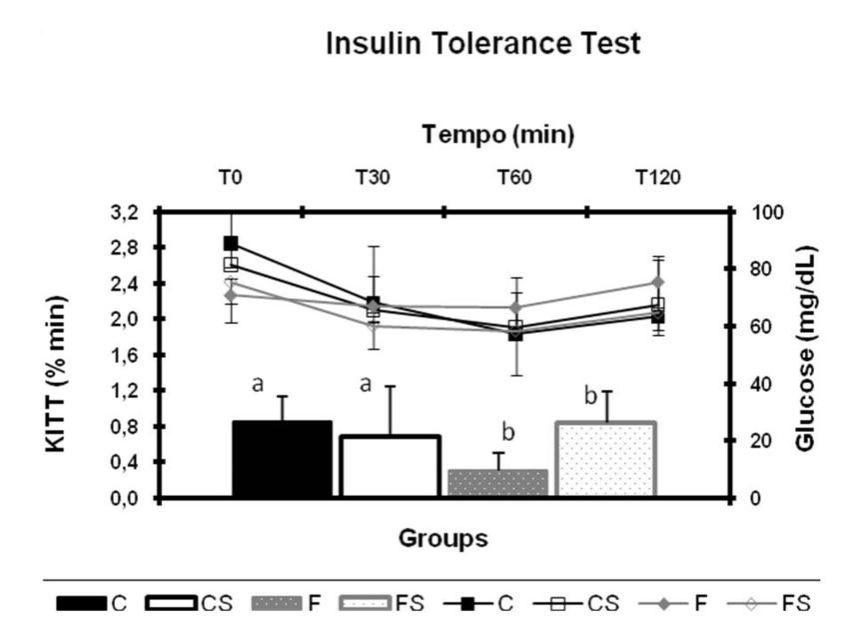
**Glucose Kinetics and Serum Glucose Removal Rate (Kitt) during ITT** (A) Serum glucose (mg/dl) and (B) serum glucose removal rate (KITT) during the insulin tolerance test. C = balanced diet, adequate litter; CS = balanced diet, small litter, F = fructose-rich diet, adequate litter, FS = high-fructose diet, small litter. Results are mean ± SD of 7 animals per group. Different letters indicate statistically significant differences (Two-way ANOVA and Newman-Keuls post hoc, P < 0.05).

Figure 5 displays the blood glucose (A) and the blood glucose removal rate (KITT) (B) during insulin tolerance test. The F group showed significantly lower values of KITT than the others, indicating insulin resistance.

**Figure 6:**
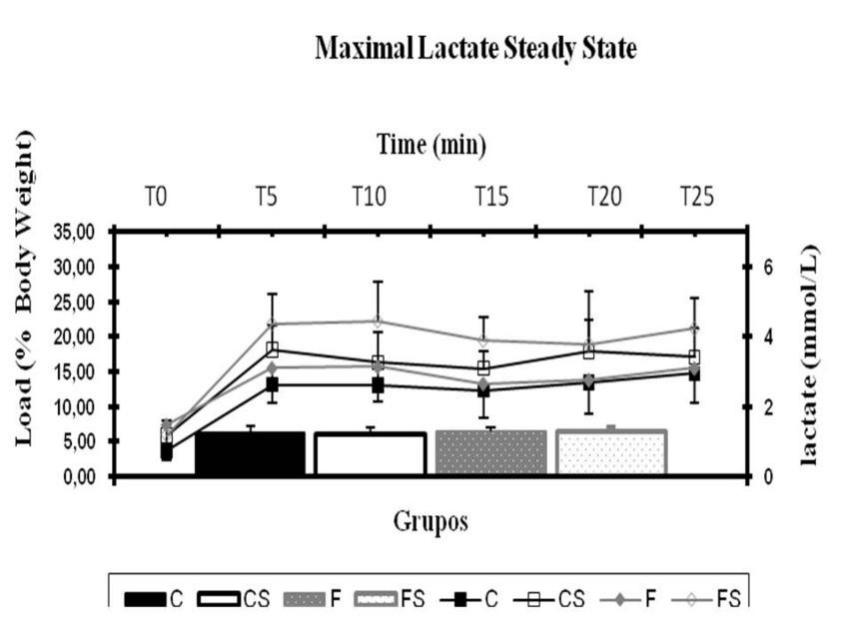
**Overload and Blood Lactate Concentration of MLSS** (A) Overload and (B) blood lactate concentration corresponding to the Maximal Lactate Steady State. C = balanced diet, adequate litter; CS = balanced diet, small litter, F = fructose-rich diet, adequate litter, FS = high-fructose diet, small litter. Results are mean ± SD of 7 animals per group.

Figure 6 shows the mean load (A) and the mean blood lactate concentration corresponding to maximal lactate steady state (B) for the different groups. There was no statistical difference between the groups.

**Figure 7:**
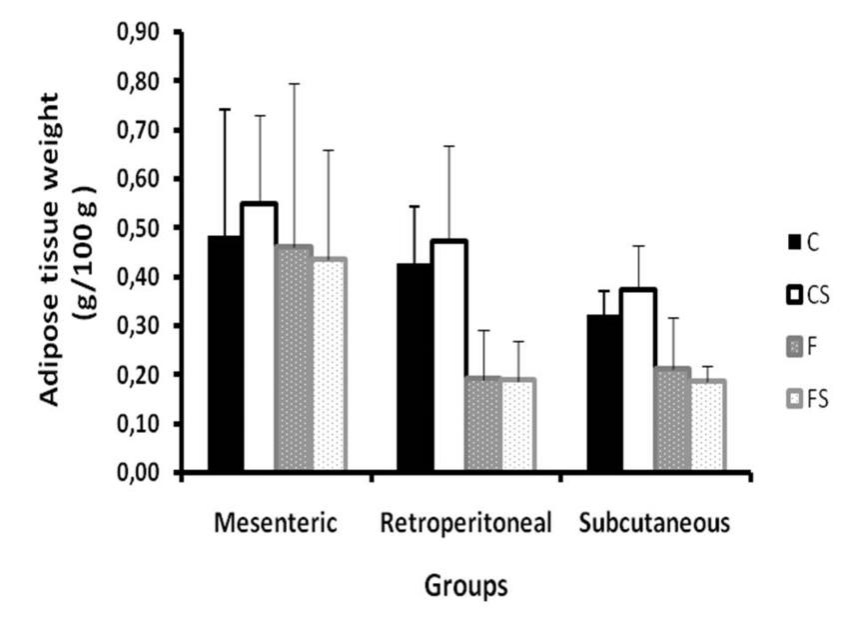
**Weight of Mesenteric, Retroperitonial and Subcutaneous Adipose Tissue** Adipose tissue weight (g/100 g) of the mesenteric, retroperitoneal and posterior subcutaneous regions. C = balanced diet, adequate litter; CS = balanced diet, small litter, F = fructose-rich diet, adequate litter, FS = high-fructose diet, small litter. Results are mean ± SD of 7 animals per group.

**Figure 8:**
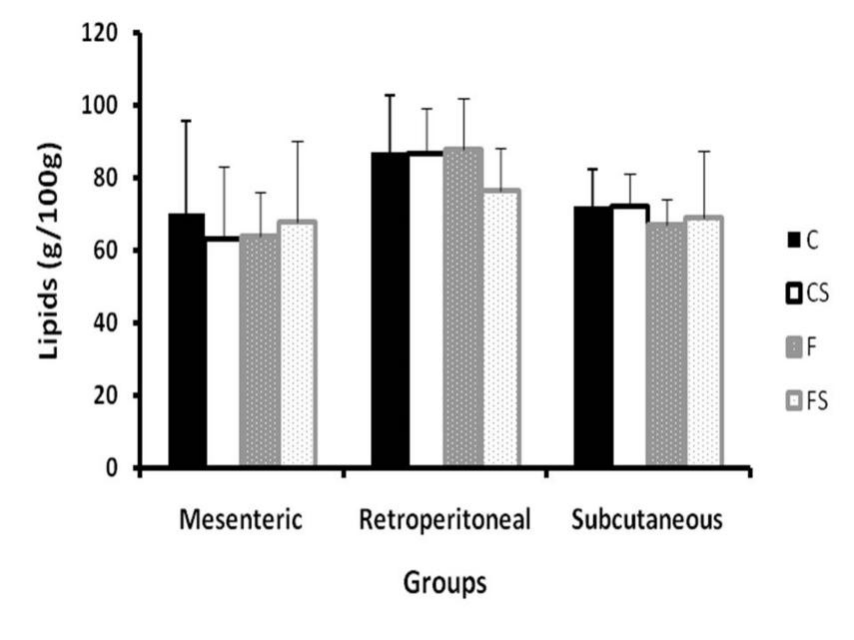
**Lipid Concentration in Mesenteric, Retroperitonial and Subcutaneous Adipose Tissue** Total lipid concentration (g/100 g) in adipose tissue of the mesenteric, retroperitoneal and subcutaneous regions. C = balanced diet, adequate litter; CS = balanced diet, small litter, F = fructose-rich diet, adequate litter, FS = high-fructose diet, small litter. Results are mean ± SD of 7 animals per group.

In the Figure 7 the values of adipose tissue weight for the mesenteric, retroperitoneal and subcutaneous regions are presented, while Figure 8 reveals the values for fat content in the same adipose tissue deposits. No significant difference between the groups was found.

The serum parameters for the groups at the end of the experiment are presented in Table 2. There were no significant differences in glucose and LDL cholesterol values between the groups but for total cholesterol it can be observed that the FS group showed statistically higher values than C and CS groups. It was observed that the F and FS groups showed significantly higher triglycerides concentrations than the C group. It was also observed significantly higher values of HDL in the groups fed the fructose-rich diet than in C group.

**Table 2:** Serum glucose (mg/dl), total cholesterol (mg/dl), HDL (mg/dl), LDL (mg/dl) e triglycerides (mg/dl) at 90 days.

Parameters	Groups			
	
	C	CS	F	FS
Glucose	85 ± 14	106 ± 28	117 ± 47	114 ± 31
Total Cholesterol	53 ± 2^a^	59 ± 7^a^	69 ± 21^ab^	80 ± 22^b^
HDL Cholesterol	24 ± 5^a^	31 ± 8^ab^	36 ± 8^b^	39 ± 15^b^
LDL Cholesterol	34 ± 5	38 ± 4	33 ± 5	37 ± 5
Triglycerides	42 ± 10^a^	78 ± 33^ab^	118 ± 66^b^	120 ± 39^b^

Results expressed as mean ± SD of 7 animals per group. Different letters indicate significantly different values. C = balanced diet, adequate litter; CS = balanced diet, small litter, F = fructose-rich diet, adequate litter, FS = high-fructose diet, small litter. Different letters indicate statistically significant differences (Two-way ANOVA and Newman-Keuls post hoc, P < 0.05).

There were no significant differences in glucose and LDL cholesterol values between the groups but for total cholesterol it can be observed that the FS group showed statistically higher values than C and CS groups. It was observed that the F and FS groups showed significantly higher triglycerides concentrations than the C group. It was also observed significantly higher values of HDL in the groups fed the fructose-rich diet than in C group.

In Table 3 no significant difference was found in glycogen synthesis, glucose uptake and glucose oxidation rates by the isolated soleous muscle between rats fed the fructose-rich diet and those fed the control.

**Table 3:** Glucose oxidation, glucose uptake and glycogen synthesis by isolated soleus muscle of rats at 90 days (umol/g.60 min).

Parameters	Groups			
	C	CS	F	FS
**Glucose Oxidation**	4.91 ± 1.2	4.93 ± 1.3	5.2 ± 1.7	4.5 ± 0.9
**Glucose Uptake**	24.4 ± 12.3	23.2 ± 10.0	22.7 ± 8.0	23.0 ± 9.8
**Gycogen Synthesis**	0.8 ± 0.2	0.88 ± 0.2	0.8 ± 0.4	0.9 ± 0.2

Results expressed as mean ± SD of 7 animals per group. C = balanced diet, adequate litter; CS = balanced diet, small litter, F = fructose-rich diet, adequate litter, FS = high-fructose diet, small litter.

## Discussion

This study analyzed the effects of fructose and of the amount of food during the fetal and post natal periods on the metabolic profile and on the aerobic capacity of rats in adulthood. Much of the knowledge we have today about the etiology of metabolic syndrome refers to studies in experimental models, but few studies were successful in developing all the metabolic changes that characterize this syndrome in animals. Some studies use genetically modified animals, which show isolated changes as dyslipidemia, hypertension and obesity [33]. Fructose-rich diet has been used in several studies to develop the signs of the metabolic syndrome for further studies of this disease that has affected millions of people around the world [11,12].

Increased amounts of fructose (group F) and/or food (groups FS and CS) administered during the fetal and postnatal periods did not change food intake of the animals when compared to controls (group C). Similar findings were reported by Moura [34]. These authors found that early administration (after weaning) of high amounts of fructose in drinking water or food did not alter food intake of rats. Increased amounts of fructose and/or food during fetal and postnatal periods did not affect the linear growth of the animals. These results also are consistent with those of Choi [35] observed in animals fed fructose after weaning.

The FS group showed weight loss when compared with C and CS groups and this loss was even greater in the F group that ingested fructose and was kept in proper litters. On the other hand, Kelley [17] and Bezerra [12] found no difference in body weight of animals fed with fructose, but these studies differ from the present study, since here the administration of fructose was made during the fetal/neonatal life, an extremely important stage for growth and development.

One aspect that deserves attention is that, at the moment of sacrifice, it was observed that the stomach and intestines of some animals fed the fructose-rich diet appeared dilated, apparently containing large amounts of gases. Symptoms such as increased diameter of the abdominal area and abdominal pain are signals present in fructose intolerance [35]. It was reported that under normal conditions, in the prenatal and suckling periods of rat development, intestinal GLUT5 (the fructose transporter at the apical cell membrane) mRNA levels and fructose transport rates are very low [36]. Therefore, the administration of large amounts of fructose during the fetal/neonatal period may lead to intolerance to the substrate, which may impair body weight gain.

The glucose tolerance of the animals was not altered by the excessive intake of fructose. However, insulin sensitivity decreased in the group fed the fructose-rich diet and maintained in proper litters (F group) during lactation. This contrasts with the study by Moura et al. [34], in which a fructose-rich diet, administered after weaning, made the animals intolerant to glucose without changing insulin sensitivity. Moreover, similar to what occurred in the present study, Bezerra [12] found insulin resistance, determined by the KITT during an insulin tolerance test.

The aerobic capacity of the animals determined by the maximal lactate steady state protocol was not affected by the ingestion of fructose, because there was no statistical difference between the animals fed fructose and balanced diet neither in the work load nor in the blood lactate concentration at the MLSS intensity during swimming exercise, in accordance with previous studies using young (weaned) rats subjected to high fructose diets [37]. Therefore, the fructose-rich diet does not impair the ability to perform aerobic exercises. This was further reinforced by the fact that, in the skeletal muscle, neither glucose oxidation nor glucose uptake and glycogen synthesis rates were also affected by the fructose-rich diet early administration.

The accumulation of adipose tissue in the mesenteric, retroperitoneal and subcutaneous regions was not affected by the fructose-rich diet. In studies with Sprague-Dowley adult rats receiving a fructose solution combined with standard diet, there was a higher body weight gain and weight of fat tissues than in the control group fed only the standard diet [38]. Therefore, an important factor, when dealing obesity/metabolic syndrome in experimental rat models, is the animal strain, as there are animals that are more susceptible to certain metabolic disorders than others.

The effects of dietary fructose on lipoprotein metabolism is an important issue, and the effects of dietary fructose on circulating triglyceride and on circulating total cholesterol as well as on HDL and LDL cholesterol become crucial in the evaluation of the metabolic impact of fructose. Serum concentrations of total cholesterol in the present study were significantly higher in the group that ate the fructose-rich diet and was kept in small litters (FS) compared to C group. Triglyceride concentrations showed to be increased in groups fed the fructose-rich diet (F and FS) when compared with C group. In the group fed the high- fructose diet and maintained in small litters (FS), the increase was even more pronounced. Several studies have also observed an increase in circulating triglyceride concentrations in rats subjected to elevated fructose intake, regardless time of life the diet was imposed [17,12]. Serum HDL concentrations were significantly elevated in the groups fed the fructose-rich diet (F and FS) when compared to controls. Similar feature was reported previously by Moz and colleagues [39] in adult Sprague-Dawley rats fed with various levels of fructose.

Studies have shown that one of the factors that often lead to insulin resistance is the excess of circulating non-esterified fatty acid (NEFA), which may be derived from the triglyceride-rich lipoproteins LDL and VLDL or from the adipose tissue. Because the anti-lipolytic action and the stimulation of lipoprotein lipase by insulin, resistance to this hormone contributes to the increase in lipolysis and in serum NEFA concentrations. NEFA acts in the liver in order to increase glucose and triglycerides synthesis and induces the increase of circulating LDL [40]. This would explain, at least in part, the origin of the insulin resistance and of the increased serum cholesterol and triglycerides in the present study in the rats that ingested fructose. Unfortunately, serum NEFA concentrations were not measured in the present study.

## Conclusion

In summary, feeding the fructose-rich diet during the intrauterine and the postnatal periods led to insulin resistance. When associated to increased food intake, feeding the fructose rich diet induced dyslipidemia, with increased serum concentrations of total cholesterol and triglycerides. Fructose rich diet intake did not impair either the aerobic capacity or the glucose oxidation by the skeletal muscle in the conditions of the present study. Taken together, these results show that the animal model evaluated is potentially interesting for the study the role of exercise in the metabolic syndrome.

## List of abbreviations

GTT: Oral Glucose Tolerance Test; HDL: High Density Lipoprotein; ITT: Insulin Tolerance Test; Kitt: Serum Glucose Disappearance Rate; LDL: Low Density Lipoprotein; MLSS: Maximal lactate steady state; NEFA: Non-Esterified Fatty Acid; TG: Triglycerides; VLDL: Very Low Density Lipoprotein; WHO: World Health Organization;

## Competing interests

The authors declare that they have no competing interests.

## Authors' contributions

ACG was responsible for the experimental design, data collection, statistical analysis and preparation of the manuscript. LTC was responsible for the experimental design and for data collection. CR and JDB were responsible for the data collection and the preparation of the manuscript. MARM was responsible for experimental design, coordination of research and preparation of the manuscript. All authors read and approved the final manuscript text.
